# Sex-based differences in emergency department treatment times for acute ischaemic stroke: evidence from a large Italian cohort

**DOI:** 10.1093/esj/aakag039

**Published:** 2026-05-11

**Authors:** Irene Scala, Marcello Covino, Pier Andrea Rizzo, Maurizio Bisegna, Francesca Donnarumma, Davide Della Polla, Nicola Bonadia, Anna Bersano, Paolo Calabresi, Giovanni Frisullo

**Affiliations:** Cerebrovascular Unit, Clinical Neuroscience Department, Fondazione IRCCS Istituto Neurologico Carlo Besta, via Giovanni Celoria 11, Milan 20133, Italy; Università Cattolica del Sacro Cuore, Largo Francesco Vito 1, Rome 00168, Italy; Università Cattolica del Sacro Cuore, Largo Francesco Vito 1, Rome 00168, Italy; Emergency Medicine Department, Fondazione Policlinico Universitario Agostino Gemelli IRCCS, Largo A. Gemelli 8, Rome 00168, Italy; Università Cattolica del Sacro Cuore, Largo Francesco Vito 1, Rome 00168, Italy; Department of Neuroscience, Organ Sense, and Thorax, Fondazione Policlinico Universitario Agostino Gemelli IRCCS, Largo A. Gemelli 8, Rome 00168, Italy; Università Cattolica del Sacro Cuore, Largo Francesco Vito 1, Rome 00168, Italy; Università Cattolica del Sacro Cuore, Largo Francesco Vito 1, Rome 00168, Italy; Emergency Medicine Department, Fondazione Policlinico Universitario Agostino Gemelli IRCCS, Largo A. Gemelli 8, Rome 00168, Italy; Emergency Medicine Department, Fondazione Policlinico Universitario Agostino Gemelli IRCCS, Largo A. Gemelli 8, Rome 00168, Italy; Cerebrovascular Unit, Clinical Neuroscience Department, Fondazione IRCCS Istituto Neurologico Carlo Besta, via Giovanni Celoria 11, Milan 20133, Italy; Università Cattolica del Sacro Cuore, Largo Francesco Vito 1, Rome 00168, Italy; Department of Neuroscience, Organ Sense, and Thorax, Fondazione Policlinico Universitario Agostino Gemelli IRCCS, Largo A. Gemelli 8, Rome 00168, Italy; Department of Neuroscience, Organ Sense, and Thorax, Fondazione Policlinico Universitario Agostino Gemelli IRCCS, Largo A. Gemelli 8, Rome 00168, Italy

**Keywords:** epidemiology, healthcare inequalities, sex differences, stroke, women

## Abstract

**Introduction:**

Sex-related disparities in acute ischaemic stroke (AIS) care have been widely reported. However, evidence from Italy remains limited. We aimed to evaluate sex differences in access to revascularisation treatments (RTs) and key time performance indicators in a large Italian cohort.

**Patients and methods:**

We conducted a single-centre, retrospective, observational study including all adults admitted to the emergency department of a comprehensive stroke centre in Rome between January 2015 and December 2022 for suspected stroke. Clinical and demographic characteristics, comorbidities, presenting symptoms, RTs and stroke care time metrics were collected. Multivariable logistic and linear regression models, as well as restricted cubic spline analyses, were used to assess sex-related differences in RTs and time indicators, adjusting for relevant confounders.

**Results:**

Within the 9167 patients, 44.4% had AIS, and 48.2% were women. Among patients with AIS, women were older (*P* < .001), had higher NIHSS scores at onset (*P* < .001), a greater comorbidity burden (*P* < .001) and higher in-hospital mortality (*P* = .010). No significant sex differences were observed in intravenous thrombolysis and endovascular treatment administration rates. However, median door-to-needle time was 4 min longer in women than in men (*P* = .030). After adjustment, female sex was not significantly associated with RT administration or in-hospital mortality, but remained an independent predictor of longer DNT [adjusted β −8.242; 95% CI (−15.453 to −1.031); *P* = .025].

**Discussion and conclusions:**

Although access to RTs was comparable between sexes, women experienced longer DNT, indicating persistent in-hospital barriers to timely stroke care. These findings highlight the need for targeted interventions to achieve full equity in AIS management.

## Graphical Abstract

**Graphical Abstract:**
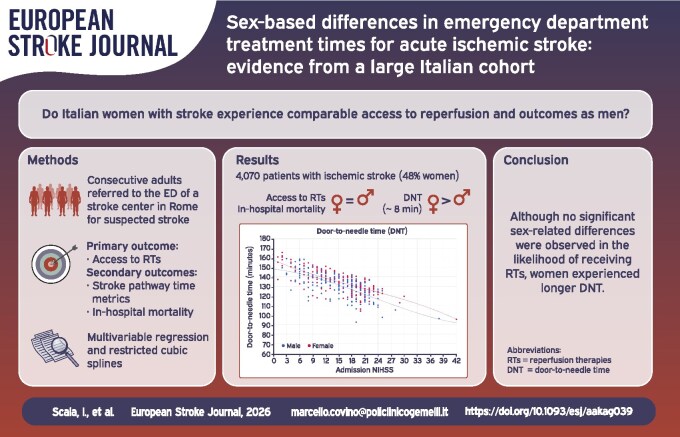

## Introduction

Ischaemic stroke remains a leading cause of mortality and disability worldwide, with its incidence continuing to rise over time.^1^ Although men have an approximately 20% higher lifetime risk of developing stroke compared to women,^2^ recent changes in lifestyle habits and population ageing have contributed to a progressive rise in stroke incidence among women in recent decades. Projections suggest that by 2030, women may surpass men in stroke incidence.^3^

Despite this trend, sex-related disparities in the early recognition, management and treatment of stroke persist. Historically, the lower burden of cardiovascular risk factors^4^ and the lower incidence of stroke among women have contributed to a reduced a priori suspicion of stroke among many emergency physicians and emergency medical service (EMS) personnel.^5,6^ This occurs even though certain stroke risk factors, such as atrial fibrillation (AF),^7^ are now more prevalent in women than in men. Furthermore, women present several sex-specific risk factors for stroke, including pregnancy, the puerperium, oestrogen–progestin therapy and hormonal influences that may predispose to stroke at a younger age.^4,8^

From a clinical perspective, women more frequently present with atypical, non-focal symptoms during stroke onset, such as altered mental status, headache or impaired consciousness, which may complicate early diagnosis.^9^ This diagnostic challenge may partially explain the longer delays in stroke recognition and treatment that have been consistently reported in women,^10^ as well as their historically lower likelihood of receiving revascularisation therapies, including intravenous thrombolysis (IVT)^11^ and endovascular treatment.^12^ Consequently, stroke outcomes tend to be worse in women compared to men,^13,14^ also considering that women often present with more severe strokes at onset^10^ and have a higher prevalence of large vessel occlusion.^10^

Given the rapid demographic and social changes occurring in high-income countries, there is an urgent need to better characterise and address sex-related disparities in stroke care. This need is particularly relevant in countries such as Italy, where data on sex differences in acute stroke management remain limited. Moreover, the persistent underrepresentation of women in stroke clinical trials raises concerns regarding the generalisability of evidence and may contribute to suboptimal treatment strategies for female patients.^15^

The primary aim of this study is to evaluate sex-related disparities in access to revascularisation treatments (RTs) for acute ischaemic stroke (AIS) in a large Italian cohort. Secondly, we aim to assess sex differences in cardiovascular risk factors, clinical presentation, stroke care time metrics and in-hospital mortality.

## Patients and methods

### Study design and population

This single-centre, retrospective, observational cohort study included adult patients admitted to the emergency department (ED) of a comprehensive stroke centre between 1 January 2015 and 31 December 2022. In accordance with the “hub-and-spoke” stroke model,^16^ our centre serves as the referral hospital for the entire northern area of the Lazio region. Given the wide catchment area of our stroke network and the widespread implementation of the hub-and-spoke model across Italy, we consider our study population to be representative of those served by other tertiary stroke centres at the regional level. However, the stroke population included in our study and the stroke pathways of our centre may differ from those of other settings, such as non-hub stroke centres and hospitals in other Italian regions, where regional practices and recommendations may vary substantially. In detail, in our ED, stroke code activation occurs immediately at triage for any patient with suspected stroke, followed by prompt evaluation by a neurologist from the dedicated stroke team and direct transfer to the CT suite for non-contrast brain CT and CT angiography of the extra- and intracranial cerebral circulation. Then, based on the multidisciplinary evaluation of the clinical history and neuroimaging findings, the vascular neurologist and the radiologist determine the patient’s eligibility for reperfusion therapies. Inclusion criteria were: (1) presentation to the ED for suspected stroke; (2) clinical evaluation in the ED by a cerebrovascular specialist and (3) age ≥ 18 years. Exclusion criteria were: (1) pregnancy; (2) insufficient or inadequate clinical information to determine the discharge diagnosis and (3) direct transfer from the ED to another hospital.

The study was conducted in accordance with the Strengthening the Reporting of Observational Studies in Epidemiology (STROBE) guidelines for observational studies.

### Data collection

Clinical and demographic data were collected anonymously and retrospectively through electronic medical record review by 3 investigators with extensive expertise in stroke management (I.S., P.A.R., M.B.).

For each patient, we collected demographic characteristics (sex and age), vital signs at the ED evaluation, presenting symptoms as documented at triage, comorbidities based on medical history and overall comorbidity burden calculated using the Charlson Comorbidity Index. In addition, laboratory parameters obtained from blood samples at the time of ED admission were collected, including complete blood count, coagulation profile, serum electrolytes, liver and renal function tests, cardiac troponins, NT-proBNP, C-reactive protein, procalcitonin and D-dimer.

The mode of patient arrival at the ED was classified as “emergency medical service” (EMS) or “walk-in.” We collected ED and in-hospital process metrics, including ED waiting time before medical assessment, triage code assignment, ED length of stay (LOS), hospitalisation rate, admission to the neurology ward and overall hospital LOS. Time elapsed from symptom onset to ED arrival (onset-to-door time, ODT) was stratified into 6 categories: < 3 h, 3–6 h, 6–12 h, 12–24 h and > 24 h.

For patients with a final diagnosis of stroke (ischaemic or haemorrhagic), the NIHSS was used to assess symptom severity at admission. For patients discharged with a diagnosis of ischaemic stroke, data on RTs were recorded. In patients who underwent RTs, several stroke care time metrics were collected, including door-to-CT scan time (DCT), door-to-needle time (DNT) and door-to-groyne time (DGT).

For each included patient, the primary discharge diagnosis was classified as stroke, stroke mimic, or transient ischaemic attack (TIA). Stroke was defined as the sudden onset of a new focal neurological deficit of vascular origin at a site consistent with the territory of a major cerebral artery and was categorised as ischaemic or haemorrhagic. The diagnosis of ischaemic stroke was based on the baseline urgent CT scan performed during the ED stay and brain MRI performed during hospitalisation, and, in patients who were ineligible for MRI, on a 24-h follow-up CT scan.

Transient ischaemic attack was defined as a transient episode of neurological dysfunction caused by focal brain, or retinal ischaemia, without acute infarction. Stroke mimics were defined as patients presenting with stroke-like symptoms and who were triaged as “suspected stroke” but in whom neither stroke nor TIA was confirmed after diagnostic work-up.

### Outcome measures

We considered RTs as the primary outcome measure for this study. Secondary outcomes included intravenous thrombolysis, endovascular treatment and stroke pathway time metrics, including DNT, DGT and DCT. In-hospital mortality was also considered as a secondary outcome measure.

### Statistical analysis

Qualitative variables were expressed as absolute and relative frequencies. The Shapiro–Wilk test was used to assess the normality of data distribution. Quantitative variables were reported as median and IQR. Comparisons between groups were performed using the χ^2^ test or Fisher’s exact test for qualitative variables, and the Mann–Whitney *U* test or Kruskal–Wallis test for quantitative variables, as appropriate.

We then performed a univariate, sex-specific comparison of the frequency of RT administration and stroke treatment time metrics in patients enrolled before and after 2018, in order to assess potential temporal changes in stroke care. We selected 2018 as the cut-off year both because it falls midway through our enrolment period and because it corresponds to the publication of the trials that extended patient eligibility for mechanical thrombectomy up to 24 h from symptom onset using perfusion imaging–based selection.

To identify independent predictors of RTs, stepwise multivariable logistic regression models were performed, adjusting for baseline NIHSS, age, sex, mode of ED arrival, assigned triage code, atrial fibrillation, onset-to-door time, arterial hypertension, major neurocognitive disorder, diabetes, cancer and history of previous stroke/TIA, based on univariate comparisons and expert opinion. For in-hospital mortality, the same variables were included with the addition of thrombolysis and/or endovascular treatment, coronary artery disease and admission to the neurology ward, considered as potential confounders based on expert opinion. Collinearity was assessed using the variance inflation factor (VIF).

To evaluate whether the probability of receiving AIS treatments differed between men and women across NIHSS scores, RCS modelling was applied. Two multivariable logistic regression models were constructed, adjusting for age and baseline NIHSS and separate odds estimates for thrombolysis and endovascular treatment were calculated for females and males based on the beta coefficients of all predictors. To describe the relationship among age, NIHSS at presentation and adjusted odds estimated by the regression models, an RCS curve was fitted using NIHSS (as a continuous variable) as the predictor and the odds of the procedure as the outcome. This non-parametric approach was chosen to allow a flexible representation of the relationship without imposing a specific parametric form a priori. Knots were placed at the 25th, 50th and 75th percentiles of the NIHSS distribution, following standard recommendations for spline modelling given the sample size.

To explore the association among age, sex, NIHSS at presentation and time to either thrombolysis or thrombectomy, we applied a multivariable linear regression model incorporating these variables. A stepwise forward selection approach was used, with removal based on the Akaike information criterion. An RCS curve was also fitted to describe the relationship between NIHSS at presentation and adjusted time values derived from the coefficients of significant predictors. As in the previous analysis, knots were placed at the 25th, 50th and 75th percentiles of the NIHSS distribution. Missing data were not imputed. All statistical analyses were performed using SPSS software v26® (IBM, Armonk, NY, USA) and custom programming in Python 3.13. A 2-sided *P*-value < .05 was considered statistically significant for all tests.

## Results

### Overall population

Among the 9167 patients included in the study, 4070 (44.4%) were discharged with a diagnosis of ischaemic stroke, 882 (9.6%) with TIA and 607 (6.6%) with intracerebral haemorrhage, while more than one-third of patients (3608; 39.4%) were ultimately diagnosed with stroke mimics. Discharge diagnoses differed significantly between sexes: ischaemic stroke and intracerebral haemorrhage were more frequent in men than in women (45.3% vs 43.5% and 7.7% vs 5.5%, respectively), while stroke mimics were more commonly observed in women (*P* < .001).

Women were significantly older than men (*P* < .001) and more frequently arrived at the ED via emergency medical service (*P* = .022). No significant sex differences were observed in onset-to-door time.

Clinical presentation at ED arrival differed substantially between sexes. Men more frequently presented with motor impairment (*P* < .001), gait disturbances (*P* < .001) and dizziness (*P* = .010), whereas females more frequently exhibited aphasia (*P* = .006), headache (*P* < .001) and impaired consciousness (*P* = .006). Vital signs at presentation also differed: women had lower median systolic and diastolic blood pressure (*P* = .016 and *P* < .001, respectively) but higher heart rate compared to men (*P* < .001).

Comorbidity profiles varied significantly by sex. Men had a higher prevalence of coronary artery disease and diabetes (both *P* < .001), as well as HIV-positive status (*P* = .020), whereas females more often had atrial fibrillation, heart failure and major neurocognitive disorder (all *P* < .001).

No significant differences were observed in assigned triage codes or median ED waiting time before medical assessment. However, a higher proportion of women waited longer than 15 min for initial evaluation (*P* = .030) and were less frequently admitted from the ED to a hospital ward (*P* < .001), including the neurology ward (*P* < .001). Additionally, women had longer ED stays (*P* < .001) but shorter overall hospital stays compared to men (*P* = .008). All the details are available in Table 1.

**Table 1:** Baseline characteristics for all patients of the study cohort.

			All (*n* = 9167)	Male (*n* = 4657)	Female (*n* = 4510)	*P*-value
**Demographics**	Age (years)		75.0 (61.0–83.0)	72.0 (60.0–81.0)	77.0 (63.0–85.0)	**<.001**
**Triage**	Triage code	Emergency	3651 (39.8%)	1859 (39.9%)	1792 (39.7%)	.983
		Urgency	4238 (46.2%)	2149 (46.1%)	2089 (46.3%)	
		Minor urgency	1278 (13.9%)	649 (13.9%)	629 (13.9%)	
	ED waiting time before medical assessment (min)		10.0 (5.0–27.0)	10.0 (5.0–26.0)	10.0 (5.0–27.0)	.078
	ED waiting time > 15 min		3481 (38.0%)	1718 (36.9%)	1763 (39.1%)	**.030**
	ED length of stay (h)		7.0 (3.0–21.6)	6.7 (2.8–20.8)	7.4 (3.2–22.3)	**<.001**
**Mode of ED arrival**	Emergency medical service		4832 (52.7%)	2400 (51.5%)	2432 (53.9%)	**.022**
**Onset to door times**	<3 h		4585 (50.0%)	2355 (50.6%)	2230 (49.4%)	.309
	3–6 h		1685 (18.4%)	845 (18.1%)	840 (18.6%)	
	6–12 h		873 (9.5%)	422 (9.1%)	451 (10.0%)	
	12–24 h		584 (6.4%)	312 (6.7%)	272 (6.0%)	
	>24 h		1440 (15.7%)	723 (15.5%)	717 (15.9%)	
**Vitals (ED admission)**	Heart rate (bpm)		80.0 (70.0–92.0)	80.0 (70.0–90.0)	81.0 (72.0–93.0)	**<.001**
	Systolic blood pressure (mmHg)		146.0 (130.0–165.0)	147.0 (130.0–166.0)	145.0 (130.0–165.0)	**.016**
	Diastolic blood pressure (mmHg)		84.0 (73.0–95.0)	85.0 (75.0–95.0)	81.0 (70.0–93.0)	**<.001**
	SaO_2_ (%)		97.0 (95.0–98.0)	97.0 (95.0–98.0)	97.0 (95.0–98.0)	.687
**Onset symptoms**	Speech disturbances		4820 (52.6%)	2383 (51.2%)	2437 (54.0%)	**.006**
	Motor impairment		5418 (59.1%)	2866 (61.5%)	2552 (56.6%)	**<.001**
	Sensory impairment		1057 (11.5%)	550 (11.8%)	507 (11.2%)	.394
	Headache		1124 (12.3%)	500 (10.7%)	624 (13.8%)	**<.001**
	Epileptic seizure		676 (7.4%)	346 (7.4%)	330 (7.3%)	.837
	Confusion/Disorientation		1920 (20.9%)	940 (20.2%)	980 (21.7%)	.069
	Impaired consciousness		1265 (13.8%)	597 (12.8%)	668 (14.8%)	**.006**
	Dizziness		735 (8.0%)	407 (8.7%)	328 (7.3%)	**.010**
	Malaise		1342 (14.6%)	653 (14.0%)	689 (15.3%)	.089
	Gait disturbances		702 (7.7%)	411 (8.8%)	291 (6.5%)	**<.001**
	Syncope		913 (10.0%)	471 (10.1%)	442 (9.8%)	.616
**Comorbidities**	Charlson Comorbidity Index		3.0 (2.0–5.0)	3.0 (2.0–5.0)	3.0 (2.0–5.0)	.131
	Previous AMI or CAD		2278 (24.9%)	1267 (27.2%)	1011 (22.4%)	**<.001**
	Atrial fibrillation		1482 (16.2%)	628 (13.5%)	854 (18.9%)	**<.001**
	Heart failure		2542 (27.7%)	1213 (26.0%)	1329 (29.5%)	**<.001**
	Arterial hypertension		3811 (41.6%)	1957 (42.0%)	1854 (41.1%)	.375
	Peripheral artery disease		1607 (17.5%)	848 (18.2%)	759 (16.8%)	.082
	Previous TIA/Stroke		3559 (38.8%)	1834 (39.4%)	1725 (38.2%)	.266
	Major neurocognitive disorder		532 (5.8%)	195 (4.2%)	337 (7.5%)	**<.001**
	COPD		380 (4.1%)	199 (4.3%)	181 (4.0%)	.533
	Liver disease		114 (1.2%)	64 (1.4%)	50 (1.1%)	.251
	Diabetes		1434 (15.6%)	831 (17.8%)	603 (13.4%)	**<.001**
	Kidney failure		2103 (22.9%)	1093 (23.5%)	1010 (22.4%)	.221
	Cancer		618 (6.7%)	316 (6.8%)	302 (6.7%)	.865
	HIV+		21 (0.2%)	16 (0.3%)	5 (0.1%)	**.020**
**Outcomes**	Hospitalisation		6190 (67.5%)	3238 (69.5%)	2952 (65.5%)	**<.001**
	Hospitalisation in neurology department		3314 (36.2%)	1793 (38.5%)	1521 (33.7%)	**<.001**
	Hospitalisation length (days)		5.4 (0.8–10.5)	5.6 (1.0–10.5)	5.2 (0.7–10.5)	**.008**
	Death		831 (9.1%)	424 (9.1%)	407 (9.0%)	.894
**Diagnosis**	Ischaemic stroke		4070 (44.4%)	2110 (45.3%)	1960 (43.5%)	**<.001**
	Brain haemorrhage		607 (6.6%)	359 (7.7%)	248 (5.5%)	
	TIA		882 (9.6%)	445 (9.6%)	437 (9.7%)	
	Mimics		3608 (39.4%)	1743 (37.4%)	1865 (41.4%)	

Abbreviations: AMI = acute myocardial infarction; bpm = beats per minutes; CAD = coronary artery disease; COPD = chronic obstructive pulmonary disease; ED = emergency department; h = hours; HIV = human immunodeficiency virus; min = minutes; mmHg = millimetres of mercury; SaO_2_ = oxygen saturation; TIA = transient ischaemic attack. Statistically significant results are reported in bold.

There were striking differences between sexes in laboratory parameters at the time of ED admission. The complete list of laboratory parameters and the comparisons are available in Table S1.

### Patients with discharge diagnoses other than ischaemic stroke

At the end of the diagnostic process, 3608 patients (39.4%) had a diagnosis of stroke mimics and 607 (6.6%) of intracerebral haemorrhage. Detailed clinical characteristics of the 2 subpopulations and a comparison between sexes are available in the supplementary materials (Tables S2 and S3, respectively).

### Patients with ischaemic stroke

Among the 4070 patients with ischaemic stroke, 1960 (48.2%) were women. Even within this subgroup, men were significantly younger than women (*P* < .001).

Regarding presenting symptoms, women more frequently exhibited speech disturbances (*P* = .001) and impaired consciousness (*P* < .001), whereas men more often presented with motor and sensory deficits (both *P* < .001), gait disturbances (*P* < .001), headache (*P* = .041) and dizziness (*P* = .023). Overall, women had more severe strokes, as reflected by higher median NIHSS scores at onset (*P* < .001). At triage, men had lower heart rate values and higher diastolic blood pressure and oxygen saturation values compared to women (all *P* < .001).

Likely due to their higher median age, women had a significantly greater comorbidity burden, as indicated by higher median Charlson Comorbidity Index scores (*P* < .001). Specifically, women had a higher prevalence of atrial fibrillation and major neurocognitive disorder (both *P* < .001), whereas men more frequently had coronary artery disease (*P* < .001), diabetes (*P* = .004), HIV-positive status (*P* = .031) and kidney failure (*P* = .046).

Regarding triage parameters, women were more often classified with an “emergency” code and less frequently with “urgency” or “minor urgency” codes compared to men (*P* = .007). Women also arrived at the ED more frequently via EMS (*P* < .001), although onset-to-door time did not differ significantly between sexes.

Overall, 934 patients (22.9%) underwent revascularisation treatments: 645 (15.8%) received intravenous thrombolysis, and 466 (11.4%) underwent endovascular treatment. No significant differences were observed between sexes in the proportion of revascularisation treatments, ED waiting time before medical assessment, DCT or DGT. However, women had significantly longer DNT than men (*P* = .030).

Although overall hospitalisation rates did not differ significantly between sexes, men had shorter ED stays (*P* = .003) and were more likely to be admitted to the neurology ward (*P* < .001) compared to women. Finally, women had higher in-hospital mortality rates than men (*P* = .010).

As for the overall study population, laboratory parameters differed significantly between sexes. Women had significantly lower haemoglobin levels and creatinine levels than men (both *P* < .001), but higher inflammation parameters, including CRP (*P* = .034), platelet count level (*P* < .001) and fibrinogen (*P* < .001). The detailed results of the clinical data in the subpopulation of patients with ischaemic stroke and a comparison between sexes are available in Table 2, whereas the complete list of laboratory parameters is available in Table S4.

**Table 2:** Baseline characteristics of patients with confirmed ischaemic stroke.

			Patients with AIS (*n* = 4070)	Males (*n* = 2110)	Females (*n* = 1960)	*P*-value
**Demographics**	Age (years)		77.0 (66.0–84.0)	74.0 (63.0–81.0)	80.0 (71.0–87.0)	**<.001**
**Triage**	Triage code	Emergency	2068 (50.8%)	1026 (48.6%)	1042 (53.2%)	**.007**
		Urgency	1639 (40.3%)	877 (41.6%)	762 (38.9%)	
		Minor urgency	363 (8.9%)	207 (9.8%)	156 (8.0%)	
	ED waiting time before medical assessment (min)		8.0 (4.0–19.0)	8.0 (4.0–20.0)	8.0 (4.0–18.0)	.183
	ED waiting time > 15 min		1211 (29.8%)	639 (30.3%)	572 (29.2%)	.443
**Mode of ED arrival**	Emergency medical service		2443 (60.0%)	1171 (55.5%)	1272 (64.9%)	**<.001**
**Onset to door times**	<3 h		1975 (48.5%)	1013 (48.0%)	962 (49.1%)	.074
	3–6 h		799 (19.6%)	401 (19.0%)	398 (20.3%)	
	6–12 h		413 (10.1%)	204 (9.7%)	209 (10.7%)	
	12–24 h		276 (6.8%)	161 (7.6%)	115 (5.9%)	
	>24 h		607 (14.9%)	331 (15.7%)	276 (14.1%)	
**ED time metrics**	ED length of stay (h)		4.9 (1.9–20.0)	4.6 (1.9–17.1)	5.2 (2.0–22.0)	**.003**
	Door-to-CT scan time (min)		22.0 (14.0–35.0)	21.5 (13.3–33.0)	22.0 (14.0–36.0)	.238
	Door-to-needle time (min)		52.0 (39.0–68.0)	50.0 (39.0–65.0)	54.0 (40.0–71.3)	**.030**
	Door-to-groyne time (min)		125.0 (101.0–150.8)	124.0 (97.5–146.5)	126.0 (102.0–158.0)	.495
**Reperfusion therapies**	Intravenous thrombolysis		645 (15.8%)	336 (15.9%)	309 (15.8%)	.890
	Endovascular treatment		466 (11.4%)	224 (10.6%)	242 (12.3%)	.083
	At least one reperfusion treatment		934 (22.9%)	470 (22.3%)	464 (23.7%)	.289
**Vitals (ED admission)**	Heart rate (bpm)		80.0 (70.0–90.0)	80.0 (70.0–90.0)	80.0 (70.0–92.0)	**<.001**
	Systolic blood pressure (mmHg)		150.0 (130.0–169.0)	150.0 (131.0–169.0)	150.0 (130.0–169.0)	.449
	Diastolic blood pressure (mmHg)		84.0 (74.0–95.0)	85.0 (75.0–96.0)	80.0 (70.0–94.0)	**<.001**
	SaO_2_ (%)		97.0 (95.0–98.0)	97.0 (95.0–98.0)	97.0 (95.0–98.0)	**<.001**
**Neurological symptoms (ED admission)**	NIHSS		12.0 (6.3–18.0)	10.0 (6.0–17.0)	13.0 (8.0–19.0)	**<.001**
	Speech disturbances		2446 (60.1%)	1216 (57.6%)	1230 (62.8%)	**.001**
	Motor impairment		2745 (67.4%)	1478 (70.0%)	1267 (64.6%)	**<.001**
	Sensory impairment		325 (8.0%)	201 (9.5%)	124 (6.3%)	**<.001**
	Headache		288 (7.1%)	166 (7.9%)	122 (6.2%)	**.041**
	Epileptic seizure		167 (4.1%)	85 (4.0%)	82 (4.2%)	.803
	Confusion/Disorientation		662 (16.3%)	323 (15.3%)	339 (17.3%)	.086
	Impaired consciousness		626 (15.4%)	262 (12.4%)	364 (18.6%)	**<.001**
	Dizziness		241 (5.9%)	142 (6.7%)	99 (5.1%)	**.023**
	Malaise		492 (12.1%)	254 (12.0%)	238 (12.1%)	.918
	Gait disturbances		326 (8.0%)	201 (9.5%)	125 (6.4%)	**<.001**
	Syncope		266 (6.5%)	150 (7.1%)	116 (5.9%)	.125
**Comorbidities**	Charlson Comorbidity Index		4.0 (3.0–6.0)	4.0 (2.0–6.0)	5.0 (3.0–6.0)	**<.001**
	Previous AMI or CAD		1021 (25.1%)	595 (28.2%)	426 (21.7%)	**<.001**
	Atrial fibrillation		1016 (25.0%)	398 (18.9%)	618 (31.5%)	**<.001**
	Heart failure		934 (22.9%)	459 (21.8%)	475 (24.2%)	.060
	Arterial hypertension		2074 (51.0%)	1069 (50.7%)	1005 (51.3%)	.696
	Peripheral artery disease		984 (24.2%)	504 (23.9%)	480 (24.5%)	.653
	Previous TIA/Stroke		2592 (63.7%)	1335 (63.3%)	1257 (64.1%)	.568
	Major neurocognitive disorder		211 (5.2%)	73 (3.5%)	138 (7.0%)	**<.001**
	COPD		179 (4.4%)	90 (4.3%)	89 (4.5%)	.669
	Liver disease		39 (1.0%)	21 (1.0%)	18 (0.9%)	.801
	Diabetes		746 (18.3%)	422 (20.0%)	324 (16.5%)	**.004**
	Kidney failure		627 (15.4%)	348 (16.5%)	279 (14.2%)	**.046**
	Cancer		280 (6.9%)	145 (6.9%)	135 (6.9%)	.984
	HIV +		5 (0.1%)	5 (0.2%)	0 (0.0%)	**.031**
**Outcomes**	Hospitalisation		3661 (90.0%)	1901 (90.1%)	1760 (89.8%)	.751
	Hospitalisation in neurology department		2318 (57.0%)	1259 (59.7%)	1059 (54.0%)	**<.001**
	Hospitalisation length (days)		7.5 (4.5–13.0)	7.4 (4.6–12.5)	7.6 (4.4–13.3)	.619
	Death		477 (11.7%)	221 (10.5%)	256 (13.1%)	**.010**

Abbreviations: AMI = acute myocardial infarction; bpm = beats per minutes; CAD = coronary artery disease; COPD = chronic obstructive pulmonary disease; ED = emergency department; h = hours; HIV = human immunodeficiency virus; min = minutes; mmHg = millimetres of mercury; SaO_2_ = oxygen saturation; TIA = transient ischaemic attack. Statistically significant results are reported in bold.

### Multivariable analyses of patients with ischaemic stroke

The results of the univariate comparisons of the clinical parameters of patients who underwent revascularisation procedures and those who did not are available in Table S5.

A univariate comparison of stroke care metrics, including RTs and time metrics, was performed separately for patients enrolled until and after 2018. In this analysis, DNT was significantly longer in women than in men only in the period after 2018 (*P* = .032), but not in the earlier period (*P* = .368). Additionally, after 2018, men underwent EVT significantly less frequently than women (*P* = .029). Details are available in Table S6.

We then ran adjusted analyses to verify whether sex was an independent predictor of differences in the likelihood of receiving RTs for ischaemic stroke or in stroke pathways time indicators in the whole population of patients with ischaemic stroke. In these analyses, sex was not an independent predictor for RTs, even when considering thrombolysis and endovascular treatment alone. However, male sex was an independent predictor of lower DNT [β −8.242, 95% CI (−15.453 to −1.031); *P* = .025]. No effect of sex was found in DGT and DCT. Please refer to Table 3 and Tables S7–S11 for the extended results of the adjusted analysis.

**Table 3:** Results of the multivariable logistic analyses and linear analyses adjusted for all relevant confounders.

Parameter	OR (95%CI)	*Β* (95%CI)	*P*-value
**Thrombolysis**	1.193 (0.837–1.700)		.330
**Endovascular treatment**	0.889 (0.640–1.237)		.486
**Revascularisation treatments**	0.464 (0.095–2.267)		.343
**Door-to-CT scan time**		−2.959 (−6.560–0.643)	.107
**Door-to-needle time**		−8.242 (−15.453–−1.031)	**.025**
**Door-to-groyne time**		−0.127 (−11.991–11.737)	.983

Abbreviations: ED = emergency department; OR = odds ratio; TIA = transient ischaemic attack.

All the analyses are adjusted for age, baseline NIHSS, mode of ED arrival, onset-to-door time, assigned triage code, atrial fibrillation, arterial hypertension, major neurocognitive disorder, diabetes, cancer, previous stroke/TIA. Statistically significant results are reported in bold.

Conversely, only atrial fibrillation (*P* = .004), hypertension, age and NIHSS at onset (all *P* < .001) emerged as independent predictors of in-hospital mortality, whereas sex did not [OR 0.834 (95% CI, 0.482–1.441); *P* = .514]. For extended results of the multivariable logistic regression analysis for in-hospital mortality, please refer to Table S12.

### Analysis of the probability of receiving RTs and stroke time metrics across different NIHSS

**Figure 1:**
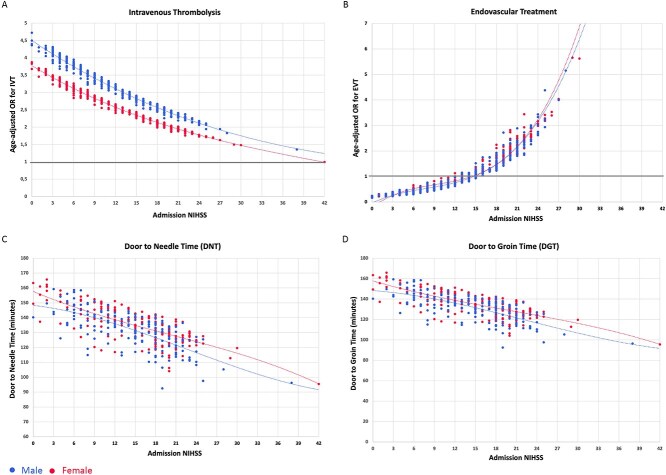
Spline curves depicting age-adjusted odds ratios for receiving IVT (panel A) and EVT (panel B) across different NIHSS values in women and men. Panels C and D show age-adjusted door-to-needle time and DGT, respectively, across NIHSS values. Abbreviations: DGT = door-to-groyne time; IVT = intravenous thrombolysis.

We then calculated the spline curves to analyse age-adjusted disparities in stroke care across different NIHSS (Figure 1). We observed that, although there were no significant sex-related differences in the OR estimates of IVT and EVT across different NIHSS values, the ORs of receiving thrombolysis was lower for women than for men at all NIHSS scores considered (Figure 1, panel A). Similarly, women had longer DNT and DGT than men for each single NIHSS score analysed, with the difference being statistically significant for DNT (Figure 1, panel C).

## Discussion

The results of our study confirm that women present with more severe strokes and that several sex-related differences in symptom onset exist. Furthermore, although no significant differences were observed between sexes in the likelihood of receiving RTs for AIS, our findings suggest that disparities in access to timely stroke care may still persist, as reflected by longer DNTs in women. No differences in in-hospital mortality rates were detected. To our knowledge, this is the first study to analyse sex-related barriers to access to RTs in such detail in Italy, and one of the first in Europe, where universal, tax-funded healthcare systems are in place, thereby providing a broader perspective on the current extent and nature of sex disparities in stroke care.

Differences in stroke severity and age at onset between sexes have been widely reported, and our results are consistent with previous findings. In our cohort, women presented with more severe strokes, reflected by higher median NIHSS scores and were significantly older, both observations consistent with prior studies.^17,18^ Regarding cardiovascular risk factors and comorbidities, women had a significantly higher overall comorbidity burden, as indicated by higher median CCI scores. In particular, consistent with previous literature,^7,18–21^ women with AIS had nearly twice the prevalence of atrial fibrillation and major neurocognitive disorders compared with men, likely explained by the strong age dependency of these conditions. Conversely, men exhibited higher prevalence of traditional cardiovascular risk factors, such as coronary artery disease and diabetes, as expected.^7,18,20,21^

With respect to access to stroke care, the absence of sex-related differences in the probability of receiving RTs aligns with recent registry-based studies reporting no significant differences between women and men in IVT and/or EVT administration rates.^17,20^ These findings may reflect growing awareness of sex disparities in healthcare, coupled with the rising incidence of stroke among women, which may have facilitated stroke recognition and is progressively narrowing historical gaps in access to RTs. This contrasts with earlier studies highlighting poorer access to RTs for women^11^ and is further supported by nationwide registry data showing that the lower likelihood of women receiving IVT observed in previous decades is no longer evident in recent years.^7,22^ Notably, the only Italian study published in 2012 on a cohort of 1272 stroke patients did not identify sex-related differences in IVT rates, consistent with our findings.^23^ However, a recent German study still reports lower RT administration rates in women, possibly reflecting regional differences in stroke care.^21^

On the other hand, our results revealed persistent sex-related differences in the quality and timeliness of stroke care. Although no differences were observed in DGT, DNT was a median of four minutes longer in women. Moreover, in the univariate comparison, sex-related differences in DNT length emerged only for the most recent period and female sex remained an independent predictor of longer DNT after adjustment for all relevant confounders, including ODT, mode of ED arrival, major comorbidities and stroke severity. These findings are consistent with previous studies from the United States^24^ and Europe^18^ reporting longer delays in IVT administration in women also in recent times. To date, however, comparable data from Italian cohorts are lacking. The delay observed in women may be attributable to several factors, including lower a priori probability of AIS, older age, higher comorbidity burden and greater severity at presentation, all of which may prompt increased clinical caution during treatment decision-making. Additionally, the higher prevalence of AF and consequent anticoagulant use may necessitate additional laboratory testing before IVT initiation to check the coagulation status, thereby prolonging in-hospital evaluation and DNT. Nevertheless, sex-related differences in stroke time metrics remained statistically significant after adjustment for these factors, suggesting that disparities in stroke care may reflect residual inequities in the timeliness of acute stroke treatment. Furthermore, women with stroke who are significantly older at stroke onset and may therefore be more likely to live alone, which can delay the retrieval of essential pre-treatment clinical information when a caregiver is not immediately available in the ED, particularly if the patient is unconscious. However, the mechanisms underlying these disparities cannot be definitively established from our findings, largely due to the retrospective design and missing data. Importantly, the independent association between female sex and longer DNT observed in the multivariable linear regression does not exclude residual confounding and should not be interpreted as evidence of causality or as an indicator of systematic disparities in stroke management.

Although the absolute median delay of approximately 4 min in women may appear modest and may fall within the range of routine clinical variability, previous literature suggests that even short in-hospital delays may be associated with worse outcome. In a large nationwide registry study including more than 14,000 thrombolysed stroke patients, the authors demonstrated that each one-minute delay in DNT was associated with a 0.6% reduction in the odds of 90-day survival, together with increased risks of intracerebral haemorrhage and worsening functional outcomes at three months.^25^ However, the clinical relevance of such a small difference in our cohort remains uncertain, particularly because the observed DNT values in both groups were within the guideline-recommended targets, suggesting that this difference may not represent a clinically meaningful deviation from established quality benchmarks.

In our AIS cohort, women exhibited a 2.6% higher prevalence of in-hospital mortality. However, despite delays in IVT administration, older age and higher stroke severity at presentation, female sex was not an independent predictor of in-hospital mortality in the fully adjusted analysis. Previous literature has reported discordant findings on this topic, both for short-term and long-term mortality and post-stroke disability.^18,26,27^ The findings of our study, limited by the lack of detailed post-stroke functional outcome data, such as mRS scores at discharge and at 3-month follow-up, do not allow us to assess the impact of longer DNT on overall clinical outcomes in women. Although DNT was significantly longer in women, no sex-related differences were found in RT administration rates, which may partially mitigate the impact of treatment delays on in-hospital mortality. While no definitive conclusions can be drawn on patient-centred outcomes, the lack of an independent association between sex and in-hospital mortality suggests that the higher crude mortality observed in women is largely driven by differences in comorbidity burden, stroke severity and age rather than sex per se.

In a previous study on the same population, we found that patients originating from non-Western European countries had a lower likelihood of receiving RTs than other patients,^28^ further supporting the present findings and highlighting persistent healthcare inequalities based on demographic characteristics in Italy. Taken together, these studies underscore the urgent need for multicentre, nationwide research to better quantify the magnitude of this problem and inform targeted educational initiatives and national programmes aimed at reducing inequalities in stroke care, even within tax-funded universal healthcare systems.

### Study limitations and strengths

This study has several limitations. First, its single-centre design limits generalisability, as data were collected from a comprehensive stroke centre in a metropolitan area, which may not be representative of smaller hospitals. In particular, centre-specific workflows, imaging availability and decision-making pathways in such a high-volume hub centre may differ from those of smaller or non-comprehensive centres, thereby potentially limiting the generalisability of the observed sex-related differences in treatment times. Additionally, regional practices and recommendations for acute stroke care pathways may vary substantially across Italian regions. Consequently, the findings of our study may reflect local clinical practice and organisational factors rather than the overall landscape of stroke care in Italy and may not be generalisable to other hospitals. Second, the retrospective design entails missing data, which may have influenced statistical analyses and hindered the understanding of the causal mechanisms underlying the observed differences. Third, we used categorical ODTs rather than continuous measures, which may have reduced our ability to detect potential sex differences in prehospital time metrics. Additionally, a key limitation of our study is the lack of adjustment for potentially relevant factors, such as pre-stroke functional status, use of anticoagulant therapy, presence of large vessel occlusion and extension of the ischaemic lesion on initial imaging and availability of collateral history. These factors may influence physicians’ decision to perform RTs in patients with acute stroke and affect treatment times, thereby contributing to residual confounding in the observed sex-related differences in DNT. As these variables are among the strongest determinants of both treatment decisions and timing, their absence may have led to biased estimates. Finally, a further limitation is the exclusive use of in-hospital mortality as the clinical outcome measure, as the absence of standardised measures for assessing the degree of functional dependence, such as the mRS scores at discharge or follow-up, restricts the assessment of patient-centred impact and limits causal interpretation of the observed treatment delays.

However, our study also has several strengths. In particular, to the best of our knowledge, this is the second study investigating sex-related barriers to access to RTs in Italy, and the first one to analyse in-depth stroke pathway time metrics and ED-related care metrics. The provision of such detailed information allows us to offer an extensive overview of the extent and nature of sex disparities in stroke care in a country with a universal healthcare system and, consequently, to generate hypotheses regarding the underlying mechanisms, thereby laying the groundwork for the design of prospective multicentric studies through which targeted healthcare policies may be implemented. In addition, our study was based on a large number of patients enrolled during an extended time period, allowing us to provide a clear picture of sex disparities in our centre over time.

## Conclusion

In conclusion, our findings suggest that although no significant sex-related differences were observed in the likelihood of receiving RTs at our comprehensive stroke centre in Rome, a modest difference in DNT was observed, with longer treatment times in women compared to men.

While this difference remained significant after adjustment for several confounders, its clinical relevance is uncertain, as it might reflect routine clinical practice variability, and the presence of residual confounding cannot be excluded. Overall, these findings highlight a sex-related difference in process metrics, but the underlying mechanisms and their impact on patient-centred outcomes remain unclear. Further prospective, multicentric studies are needed to better characterise these differences and assess their clinical significance.

## Supplementary Material

aakag039_Supplemental_Files

## Data Availability

The data supporting the findings of this study are available from the corresponding author upon reasonable request.
