# Supplementary material for: Sex-based differences in emergency department treatment times for acute ischaemic stroke: evidence from a large Italian cohort
Source: Eur Stroke J. 2026 May 11;11(5):aakag039. doi: 10.1093/esj/aakag039 (PMC13160415; doi:10.1093/esj/aakag039)
Supplement: aakag039_Supplemental_Files [file aakag039_supplemental_files.zip › Table_S10_aakag039.docx]

**Table S10.** Results of the multivariable linear regression for Door-to-groin time.

| **Parameter** | **B (95%CI)** | **p-value** | **VIF** |
| --- | --- | --- | --- |
| Sex | -0.127 (-11.991 – 11.737) | 0.983 | 1.110 |
| Age | 0.315 (-0.176 – 0.805) | 0.208 | 1.199 |
| NIHSS | -0.502 (-1.151 – 0.147) | 0.129 | 1.087 |
| Onset to door time | 0.227 (-7.251 – 7.704) | 0.953 | 1.186 |
| Emergency Medical Service | 9.474 (-6.184 – 25.132) | 0.235 | 1.101 |
| Triage codes | 54.902 (32.823 – 76.981) | **<0.001** | 1.133 |
| Diabetes | -3.123 (-20.065 – 13.801) | 0.716 | 1.029 |
| Cancer | -10.705 (-37.805 – 16.396) | 0.438 | 1.024 |
| Arterial hypertension | 6.566 (-5.009 – 18.142) | 0.265 | 1.042 |
| Atrial fibrillation | 6.843 (-5.577 – 19.263) | 0.279 | 1.160 |
| Major neurocognitive disorder | 4.053 (-37.504 – 45.611) | 0.848 | 1.020 |
| Previous stroke/TIA | 0.120 (-12.701 – 12.941) | 0.985 | 1.023 |

*Abbreviations: OR, Odds Ratio; CI, Confidence Interval; VIF, Variance Inflation Factor; NIHSS, National Institutes of Health Stroke Scale; TIA, Transient Ischemic Attack. *reference value.*
