# Supplementary material for: Sex-based differences in emergency department treatment times for acute ischaemic stroke: evidence from a large Italian cohort
Source: Eur Stroke J. 2026 May 11;11(5):aakag039. doi: 10.1093/esj/aakag039 (PMC13160415; doi:10.1093/esj/aakag039)
Supplement: aakag039_Supplemental_Files [file aakag039_supplemental_files.zip › Table_S11_aakag039.docx]

**Table S11.** Results of the multivariable linear regression for Door-to-CT scan time.

| **Parameter** | **B (95%CI)** | **p-value** | **VIF** |
| --- | --- | --- | --- |
| Sex | -2.959 (-6.560 – 0.643) | 0.107 | 1.110 |
| Age | 0.055 (-0.088 – 0.197) | 0.452 | 1.233 |
| NIHSS | -0.132 (-0.360 – 0.095) | 0.254 | 1.152 |
| Onset to door time | 3.047 (0.599 – 5.495) | **0.015** | 1.137 |
| Emergency Medical Service | -0.069 (-4.643 – 4.504) | 0.976 | 1.126 |
| Triage codes | 35.719 (30.717 – 40.721) | **<0.001** | 1.181 |
| Diabetes | -0.696 (-5.355 – 3.963) | 0.769 | 1.048 |
| Cancer | 4.735 (-3.382 – 12.852) | 0.253 | 1.016 |
| Arterial hypertension | -0.511 (-4.092 – 3.071) | 0.780 | 1.076 |
| Atrial fibrillation | -1.620 (-5.600 – 2.359) | 0.424 | 1.166 |
| Major neurocognitive disorder | 3.942 (-7.936 – 15.819) | 0.515 | 1.026 |
| Previous stroke/TIA | -5.726 (-9.735 – 1.716) | 0.005 | 1.036 |

*Abbreviations: OR, Odds Ratio; CI, Confidence Interval; VIF, Variance Inflation Factor; NIHSS, National Institutes of Health Stroke Scale; TIA, Transient Ischemic Attack. *reference value.*
