# Supplementary material for: Sex-based differences in emergency department treatment times for acute ischaemic stroke: evidence from a large Italian cohort
Source: Eur Stroke J. 2026 May 11;11(5):aakag039. doi: 10.1093/esj/aakag039 (PMC13160415; doi:10.1093/esj/aakag039)
Supplement: aakag039_Supplemental_Files [file aakag039_supplemental_files.zip › Table_S12_aakag039.docx]

**Table S12.** Results of the multivariable linear regression for in-hospital mortality.

| **Parameter** | **OR (95%CI)** | **p-value** | **VIF** |
| --- | --- | --- | --- |
| Sex | 0.834 (0.482 – 1.441) | 0.514 | 1.129 |
| Thrombolysis | 1.135 (0.620 – 2.079) | 0.681 | 1.713 |
| Thrombectomy | 1.307 (0.617 – 2.766) | 0.484 | 2.062 |
| Admission to the Neurology ward | 1.343 (0.760 – 2.374) | 0.310 | 1.442 |
| Age | 1.075 (1.046 – 1.105) | **<0.001** | 1.251 |
| NIHSS | 1.092 (1.050 – 1.137) | **<0.001** | 1.301 |
| Onset to door time | 1.028 (0.685 – 1.544) | 0.894 | 1.215 |
| Emergency Medical Service | 0.402 (0.153 – 1.056) | 0.064 | 1.145 |
| Triage codes | 0.911 (0.361 – 2.299) | 0.843 | 1.207 |
| MI or coronary artery disease | 0.913 (0.494 – 1.687) | 0.771 | 1.180 |
| Diabetes | 0.712 (0.378 – 1.342) | 0.294 | 1.053 |
| Cancer | 1.354 (0.300 – 6.114) | 0.693 | 1.014 |
| Arterial hypertension | 2.552 (1.514 – 4.303) | **<0.001** | 1.083 |
| Atrial fibrillation | 2.337 (1.308 – 4.176) | **0.004** | 1.206 |
| Major neurocognitive disorder | 0.657 (0.150 – 2.872) | 0.577 | 1.028 |
| Previous stroke/TIA | 1.551 (0.872 – 2.760) | 0.135 | 1.229 |

*Abbreviations: OR, Odds Ratio; CI, Confidence Interval; VIF, Variance Inflation Factor; NIHSS, National Institutes of Health Stroke Scale; MI, Myocardial Infarction; TIA, Transient Ischemic Attack.*
