# Supplementary material for: Sex-based differences in emergency department treatment times for acute ischaemic stroke: evidence from a large Italian cohort
Source: Eur Stroke J. 2026 May 11;11(5):aakag039. doi: 10.1093/esj/aakag039 (PMC13160415; doi:10.1093/esj/aakag039)
Supplement: aakag039_Supplemental_Files [file aakag039_supplemental_files.zip › Table_S1_aakag039.docx]

**Table S1.** Laboratory parameters of the overall study population at the time of triage and a comparison between sexes.

|  | **All**  (n = 9167) | **Males**  (n = 4657) | **Females**  (n = 4510) | **p-value** |
| --- | --- | --- | --- | --- |
| Haemoglobin (g/dL) | 13.7 (12.3-14.8) | 14.3 (12.9-15.4) | 13.1 (11.9-14.1) | **<0.001** |
| White Blood Count (x10^9/L) | 8.5 (6.8-10.9) | 8.5 (6.9-10.9) | 8.4 (6.7-10.8) | 0.144 |
| Red Blood Count (x10^9/L) | 4.6 (4.2-5.0) | 4.7 (4.3-5.1) | 4.5 (4.1-4.8) | **<0.001** |
| Mean Corpuscular Volume (fL) | 88.4 (84.7-92.0) | 88.8 (85.3-92.6) | 87.8 (84.1.91.4) | **<0.001** |
| Platelet count (x10^9/L) | 227.0 (184.0-280.0) | 212.0 (173.0-260.0) | 243.0 (199.0-298.0) | **<0.001** |
| Neutrophils (x10^9/L) | 5.8 (4.3-8.1) | 5.8 (4.3-8.1) | 5.7 (4.2-8.1) | 0.550 |
| Lymphocytes (x10^9/L) | 1.7 (1.2-2.3) | 1.7 (1.3-2.3) | 1.7 (1.2-2.3) | 0.871 |
| Prothrombin Time (s) | 11.4 (10.9-12.1) | 11.5 (11.0-12.3) | 11.3 (10.8-11.9) | **<0.001** |
| aPTT (s) | 30.5 (27.7-34.1) | 31.3 (28.3-34.6) | 29.8 (27.2-33.4) | **<0.001** |
| INR | 1.1 (1.0-1.1) | 1.1 (1.0-1.1) | 1.0 (1.0-1.1) | **<0.001** |
| Fibrinogen (mg/dL) | 342.0 (292.0-412.0) | 336.0 (287.0-407.0) | 348.0 (299.0-417.0) | **<0.001** |
| D-dimer (ng/mL) | 927.0 (382.0-2239.5) | 1086.5 (416.3-2509.0) | 814.0 (349.0-1946.0) | **0.079** |
| Creatinine (mg/dL) | 0.9 (0.7-1.1) | 1.0 (0.8-1.2) | 0.8 (0.6-1.0) | **<0.001** |
| Sodium (mmol/L) | 140.0 (137.0-141.0) | 140.0 (138.0-141.0) | 140.0 (137.0-141.0) | **0.048** |
| Potassium (mmol/L) | 4.1 (3.8-4.5) | 4.1 (3.8-4.4) | 4.0 (3.7-4.5) | 0.201 |
| Calcium (mg/dL) | 9.5 (9.2-9.9) | 9.5 (9.2-9.8) | 9.6 (9.2-9.9) | **<0.001** |
| GPT (U/L) | 16.0 (11.0-23.0) | 18.0 (12.0-25.0) | 14.0 (11.0-21.0) | **<0.001** |
| GOT (U/L) | 20.0 (16.0-28.0) | 20.0 (16.0-29.0) | 20.0 (15.0-27.0) | **0.010** |
| Total bilirubin (mg/dL) | (0.7-0.5-0.9) | 0.7 (0.5-1.0) | 0.6 (0.5-0.8) | **<0.001** |
| C reactive Protein (mg/L) | 5.6 (1.1-24.6) | 5.3 (1.0-27.9) | 5.9 (1.2-22.0) | 0.610 |
| Procalcitonin (ng/mL) | 0.1 (0.1-0.2) | 0.1 (0.1-0.2) | 0.1 (0.1-0.2) | 0.444 |
| NT-proBNP (pg/mL) | 1143.0 (302.0-3235.0) | 1077.5 (211.3-3428.5) | 1176.0 (370.5-3200.0) | 0.257 |
| Troponin Ultra (ng/L) | 0.01 (0.01-0.03) | 0.01 (0.01-0.02) | 0.01 (0.01-0.03) | **0.030** |
