# Supplementary material for: Sex-based differences in emergency department treatment times for acute ischaemic stroke: evidence from a large Italian cohort
Source: Eur Stroke J. 2026 May 11;11(5):aakag039. doi: 10.1093/esj/aakag039 (PMC13160415; doi:10.1093/esj/aakag039)
Supplement: aakag039_Supplemental_Files [file aakag039_supplemental_files.zip › Table_S2_aakag039.docx]

**Table S2.** Clinical characteristics of patients with a discharge diagnosis of a stroke-mimicking condition.

|  | | | **Mimics patients**  (n=3608) | **Male**  (n = 1743) | **Female**  (n = 1865) | **p-value** |
| --- | --- | --- | --- | --- | --- | --- |
| **Demographics** | Age (years) | | 70.0 (54.0-81.0) | 69.0 (56.0-80.0) | 71.0 (53.0-82.0) | 0.146 |
| **Triage** | Triage code | Emergency | 955 (26.5%) | 483 (27.7%) | 472 (25.3%) | 0.250 |
|  |  | Urgency | 1900 (52.7%) | 906 (52.0%) | 994 (53.3%) |  |
|  |  | Minor Urgency | 753 (20.9%) | 354 (20.3%) | 399 (21.4%) |  |
|  | ED waiting time before medical assessment (min) | | 14.0 (7.0-40.0) | 14.0 (6.0-37.0) | 15.0 (7.0-44.0) | **0.019** |
|  | ED waiting time>15 minutes | | 1741 (48.3%) | 804 (46.1%) | 937 (50.2%) | **0.013** |
|  | ED length of stay (h) | | 8.6 (4.9-22.6) | 8.7 (4.8-22.9) | 8.4 (4.9-22.4) | 0.863 |
| **Mode of ED arrival** | Emergency Medical Service | | 1545 (42.8%) | 765 (43.9%) | 780 (41.8%) | 0.210 |
| **Onset to door times** | <3 hours | | 1712 (47.5%) | 850 (48.8%) | 862 (46.2%) | 0.094 |
|  | 3-6 hours | | 629 (17.4%) | 318 (18.2%) | 311 (16.7%) |  |
|  | 6-12 hours | | 352 (9.8%) | 162 (9.3%) | 190 (10.2%) |  |
|  | 12-24 hours | | 240 (6.7%) | 115 (6.6%) | 125 (6.7%) |  |
|  | >24 hours | | 675 (18.7%) | 298 (17.1%) | 377 (20.2%) |  |
| **Vitals**  **(ED admission)** | Heart rate (bpm) | | 82.0 (72.0-94.0) | 141.0 (126.0-160.0) | 140.0 (124.0-160.0) | **0.008** |
|  | Systolic blood pressure (mmHg) | | 140.0 (125.0-160.0) | 141.0 (126.0-160.0) | 140.0 (124.0-160.0) | 0.074 |
|  | Diastolic blood pressure (mmHg) | | 82.0 (71.0-93.0) | 84.0 (72.0-95.0) | 80.0 (70.0-91.0) | 0.011 |
|  | SaO2 (%) | | 97.0 (95.0-98.0) | 97.0 (95.0-98.0) | 97.0 (95.0-99.0) | **0.003** |
| **Neurological symptoms**  **(ED admission)** | Aphasia | | 1443 (40.0%) | 668 (38.3%) | 775 (41.6%) | **0.048** |
|  | Motor impairment | | 1739 (48.2%) | 869 (49.9%) | 870 (46.6%) | 0.054 |
|  | Sensory impairment | | 555 (15.4%) | 249 (14.3%) | 306 (16.4%) | 0.078 |
|  | Headache | | 655 (18.2%) | 254 (14.6%) | 401 (21.5%) | **<0.001** |
|  | Epileptic seizure | | 432 (12.0%) | 224 (12.9%) | 208 (11.2%) | 0.116 |
|  | Confusion/Disorientation | | 926 (25.7%) | 444 (25.5%) | 482 (25.8%) | 0.799 |
|  | Impaired consciousness | | 411 (11.4%) | 204 (11.7%) | 207 (11.1%) | 0.568 |
|  | Dizziness | | 412 (11.4%) | 221 (12.7%) | 191 (10.2%) | **0.021** |
|  | Malaise | | 643 (17.8%) | 283 (16.2%) | 360 (19.3%) | **0.016** |
|  | Gait disturbances | | 265 (7.3%) | 143 (8.2%) | 122 (6.5%) | 0.056 |
|  | Syncope | | 505 (14.0%) | 252 (14.5%) | 253 (13.6%) | 0.440 |
| **Comorbidities** | Charlson Comorbidity Index | | 2.0 (1.0-4.0) | 2.0 (1.0-4.0) | 2.0 (1.0-4.0) | 0.120 |
|  | Previous AMI or CAD | | 882 (24.4%) | 442 (25.4%) | 440 (23.6%) | 0.217 |
|  | Atrial fibrillation | | 269 (7.5%) | 122 (7.0%) | 147 (7.9%) | 0.313 |
|  | Heart failure | | 1233 (34.2%) | 548 (31.4%) | 685 (36.7%) | **0.001** |
|  | Arterial hypertension | | 1002 (27.8%) | 493 (28.3%) | 509 (27.3%) | 0.506 |
|  | Peripheral artery disease | | 342 (9.5%) | 191 (11.0%) | 151 (8.1%) | **0.003** |
|  | Previous TIA/Stroke | | 547 (15.2%) | 285 (16.4%) | 262 (14.0%) | 0.054 |
|  | Major neurocognitive disorder | | 246 (6.8%) | 96 (5.5%) | 150 (8.0%) | **0.003** |
|  | COPD | | 138 (3.8%) | 78 (4.5%) | 60 (3.2%) | **0.049** |
|  | Liver disease | | 61 (1.7%) | 37 (2.1%) | 24 (1.3%) | 0.052 |
|  | Diabetes | | 448 (12.4%) | 262 (15.0%) | 186 (10.0%) | **<0.001** |
|  | Kidney failure | | 1091 (30.2%) | 527 (30.2%) | 564 (30.2%) | 0.997 |
|  | Cancer | | 258 (7.2%) | 126 (7.2%) | 132 (7.1%) | 0.860 |
|  | Metastasis | | 65 (1.8%) | 34 (2.0%) | 31 (1.7%) | 0.515 |
|  | HIV + | | 13 (0.4%) | 8 (0.5%) | 5 (0.3%) | 0.339 |
| **Outcomes** | Hospitalization | | 1379 (38.2%) | 707 (40.6%) | 672 (36.0%) | **0.005** |
|  | Hospitalization in Neurology department | | 432 (12.0%) | 219 (12.6%) | 213 (11.4%) | 0.290 |
|  | Hospitalization length (days) | | 0.8 (0.3-6.6) | 0.9 (0.3-7.0) | 0.7 (0.3-6.4) | **0.038** |
|  | Death | | 181 (5.0%) | 99 (5.7%) | 82 (4.4%) | 0.078 |

*Abbreviations: ED, Emergency Department; min, minutes; h, hours; bpm, beats per minutes; mmHg, millimetres of mercury; SaO2, Oxygen Saturation;; AMI, Acute Myocardial Infarction; CAD, Coronary Artery Disease; TIA, Transient Ischemic Attack; COPD, Chronic Obstructive Pulmonary Disease; HIV, Human Immunodeficiency Virus*
