# Supplementary material for: Sex-based differences in emergency department treatment times for acute ischaemic stroke: evidence from a large Italian cohort
Source: Eur Stroke J. 2026 May 11;11(5):aakag039. doi: 10.1093/esj/aakag039 (PMC13160415; doi:10.1093/esj/aakag039)
Supplement: aakag039_Supplemental_Files [file aakag039_supplemental_files.zip › Table_S3_aakag039.docx]

**Table S3.** Clinical characteristics of patients with a discharge diagnosis of a Intracranial haemorrhage.

|  | | | **Brain haemorrhages**  (n=607) | **Male**  (n = 359) | **Female**  (n = 248) | **p-value** |
| --- | --- | --- | --- | --- | --- | --- |
| **Demographics** | Age (years) | |  | 73.0 (61.0-81.0) | 78.0 (65.0-84.0) | **0.001** |
| **Triage** | Triage code | Emergency | 435 (71.7%) | 261 (72.7%) | 174 (70.2%) | 0.666 |
|  |  | Urgency | 145 (23.9%) | 84 (23.4%) | 61 (24.6%) |  |
|  |  | Minor Urgency | 27 (4.4%) | 14 (3.9%) | 13 (5.2%) |  |
|  | ED waiting time before medical assessment (min) | | 6.0 (3.0-10.0) | 6.0 (3.0-10.0) | 6.0 (3.0-11.0) | 0.734 |
|  | ED waiting time>15 minutes | | 110 (18.1%) | 62 (17.3%) | 48 (19.4%) | 0.512 |
|  | ED length of stay (h) | | 6.1 (2.1-23.4) | 5.6 (2.1-20.5) | 7.4 (2.4-25.3) | 0.061 |
| **Mode of ED arrival** | Emergency Medical Service | | 466 (76.8%) | 272 (75.8%) | 194 (78.2%) | 0.481 |
| **Onset to door times** | <3 hours | | 413 (68.0%) | 252 (70.2%) | 161 (64.9%) | 0.640 |
|  | 3-6 hours | | 80 (13.2%) | 42 (11.7%) | 38 (15.3%) |  |
|  | 6-12 hours | | 35 (5.8%) | 21 (5.8%) | 14 (5.6%) |  |
|  | 12-24 hours | | 26 (4.3%) | 15 (4.2%) | 11 (4.4%) |  |
|  | >24 hours | | 53 (8.7%) | 29 (8.1%) | 24 (9.7%) |  |
| **Vitals**  **(ED admission)** | Heart rate (bpm) | | 80.0 (70.0-94.0) | 80.0 (70.0-95.3) | 82.0 (70.0-92.8) | 0.817 |
|  | Systolic blood pressure (mmHg) | | 160.0 (140.0-180.0) | 160.0 (140.0-180.0) | 160.0 (140.0-180.0) | 0.934 |
|  | Diastolic blood pressure (mmHg) | | 90.0 (80.0-100.0) | 90.0 (80.0-101.0) | 90.0 (76.0-100.0) | **0.029** |
|  | SaO2 (%) | | 97.0 (95.0-98.0) | 97.0 (95.0-98.0) | 97.0 (95.0-99.0) | 0.175 |
| **Neurological symptoms**  **(ED admission)** | Aphasia | | 351 (57.8%) | 212 (59.1%) | 139 (56.0%) | 0.461 |
|  | Motor impairment | | 382 (62.9%) | 232 (64.6%) | 150 (60.5%) | 0.299 |
|  | Sensory impairment | | 24 (4.0%) | 14 (3.9%) | 10 (4.0%) | 0.934 |
|  | Headache | | 87 (14.3%) | 44 (12.3%) | 43 (17.3%) | 0.079 |
|  | Epileptic seizure | | 35 (5.8%) | 21 (5.8%) | 14 (5.6%) | 0.915 |
|  | Confusion/Disorientation | | 102 (16.8%) | 60 (16.7%) | 42 (16.9%) | 0.943 |
|  | Impaired consciousness | | 170 (28.0%) | 105 (29.2%) | 65 (26.2%) | 0.413 |
|  | Dizziness | | 17 (2.8%) | 11 (3.1%) | 6 (2.4%) | 0.636 |
|  | Malaise | | 73 (12.0%) | 45 (12.5%) | 28 (11.3%) | 0.643 |
|  | Gait disturbances | | 29 (4.8%) | 16 (4.5%) | 13 (5.2%) | 0.656 |
|  | Syncope | | 62 (10.2%) | 33 (9.2%) | 29 (11.7%) | 0.317 |
| **Comorbidities** | Charlson Comorbidity Index | | 4.0 (2.0-5.0) | 4.0 (2.0-5.0) | 4.0 (2.0-5.0) | 0.822 |
|  | Previous AMI or CAD | | 157 (25.9%) | 101 (28.1%) | 56 (22.6%) | 0.125 |
|  | Atrial fibrillation | | 68 (11.2%) | 44 (12.3%) | 24 (9.7%) | 0.322 |
|  | Heart failure | | 137 (22.6%) | 85 (23.7%) | 52 (21.0%) | 0.433 |
|  | Arterial hypertension | | 315 (51.9%) | 188 (52.4%) | 127 (51.2%) | 0.779 |
|  | Peripheral artery disease | | 103 (17.0%) | 66 (18.4%) | 37 (14.9%) | 0.264 |
|  | Previous TIA/Stroke | | 158 (26.0%) | 92 (25.6%) | 66 (26.6%) | 0.785 |
|  | COPD | | 24 (4.0%) | 12 (3.3%) | 12 (4.8%) | 0.352 |
|  | Connective tissue disease | | 4 (0.7%) | 2 (0.6%) | 2 (0.8%) | 0.709 |
|  | Liver disease | | 6 (1.0%) | 2 (0.6%) | 4 (1.6%) | 0.196 |
|  | Diabetes | | 84 (13.8%) | 60 (16.7%) | 24 (9.7%) | **0.014** |
|  | Kidney failure | | 114 (18.8%) | 76 (21.2%) | 38 (15.3%) | 0.070 |
|  | Cancer | | 36 (5.9%) | 22 (6.1%) | 14 (5.6%) | 0.804 |
|  | Metastasis | | 8 (1.3%) | 5 (1.4%) | 3 (1.2%) | 0.846 |
|  | HIV + | | 2 (0.3%) | 2 (0.6%) | 0 | 0.239 |
| **Outcomes** | Hospitalization | | 594 (97.9%) | 351 (97.8%) | 243 (98.0%) | 0.859 |
|  | Hospitalization in Neurology department | | 248 (40.9%) | 153 (42.6%) | 95 (38.3%) | 0.288 |
|  | Hospitalization length (days) | | 9.3 (5.0-17.9) | 8.8 (4.0-18.0) | 10.0 (5.4-17.4) | 0.364 |
|  | Death | | 157 (25.9%) | 93 (25.9%) | 64 (25.8%) | 0.978 |

*Abbreviations: ED, Emergency Department; min, minutes; h, hours; bpm, beats per minutes; mmHg, millimetres of mercury; SaO2, Oxygen Saturation;; AMI, Acute Myocardial Infarction; CAD, Coronary Artery Disease; TIA, Transient Ischemic Attack; COPD, Chronic Obstructive Pulmonary Disease; HIV, Human Immunodeficiency Virus*
