# Supplementary material for: Sex-based differences in emergency department treatment times for acute ischaemic stroke: evidence from a large Italian cohort
Source: Eur Stroke J. 2026 May 11;11(5):aakag039. doi: 10.1093/esj/aakag039 (PMC13160415; doi:10.1093/esj/aakag039)
Supplement: aakag039_Supplemental_Files [file aakag039_supplemental_files.zip › Table_S4_aakag039.docx]

**Table S4.** Laboratory parameters of the subpopulation of ischemic stroke patients at the time of triage and a comparison between sexes.

|  | **Patients with AIS**  (n = 4070) | **Males**  (n = 2110) | **Females**  (n = 1960) | **p-value** |
| --- | --- | --- | --- | --- |
| Haemoglobin (g/dL) | 13.7 (12.3-14.9) | 14.3 (13.0-15.4) | 13.0 (11.7-14.1) | **<0.001** |
| White Blood Count (x10^9/L) | 8.7 (7.0-11.1) | 8.7 (7.0-10.9) | 8.8 (6.9-11.2) | 0.432 |
| Red Blood Count (x10^9/L) | 4.6 (4.2-5.0) | 4.7 (4.3-5.1) | 4.5 (4.1-4.9) | **<0.001** |
| Mean Corpuscular Volume (fL) | 88.4 (84.8-92.2) | 89.0 (85.5-92.8) | 87.8 (84.2-91.4) | **<0.001** |
| Platelet count (x10^9/L) | 225.0 (181.0-282.3) | 212.0 (171.0-261.0) | 242.0 (194.0-300.0) | **<0.001** |
| Neutrophils (x10^9/L) | 6.0 (4.5-8.4) | 5.9 (4.5-8.1) | 6.2 (4.5-8.6) | **0.011** |
| Lymphocytes (x10^9/L) | 1.7 (1.2-2.3) | 1.8 (1.3-2.3) | 1.7 (1.2-2.2) | **<0.001** |
| Prothrombin Time (s) | 11.4 (10.9-12.2) | 11.5 (11.0-12.2) | 11.4 (10.9-12.1) | **<0.001** |
| aPTT (s) | 30.3 (27.6-34.0) | 30.9 (28.2-34.4) | 29.6 (27.0-33.4) | **<0.001** |
| INR | 1.06 (1.02-1.13) | 1.07 (1.02-1.14) | 1.1 (1.0-1.1) | **<0.001** |
| Fibrinogen (mg/dL) | 349.0 (302.0-418.0) | 341.0 (293.0-411.0) | 358.0 (311.0-427.0) | **<0.001** |
| D-dimer (ng/mL) | 1336.0 (625.5-3577.3) | 2030.5 (933.3-5114.8) | 1098.5 (513.0-2696.5) | **0.034** |
| Creatinine (mg/dL) | 0.89 (0.73-1.12) | 1.0 (0.8-1.2) | 0.8 (0.7-1.0) | **<0.001** |
| Sodium (mmol/L) | 140.0 (137.0-141.0) | 140.0 (138.0-141.0) | 139.0 (137.0-141.0) | 0.480 |
| Potassium (mmol/L) | 4.1 (3.8-4.5) | 4.1 (3.8-4.5) | 4.1 (3.8-4.6) | 0.459 |
| Calcium (mg/dL) | 9.5 (9.2-9.8) | 9.5 (9.2-9.8) | 9.6 (9.2-9.9) | **<0.001** |
| GPT (U/L) | 16.0 (11.0-23.0) | 17.0 (12.0-25.0) | 14.0 (10.0-21.0) | **<0.001** |
| GOT (U/L) | 20.0 (15.0-27.0) | 20.0 (16.0-27.0) | 20.0 (15.0-26.0) | 0.570 |
| Total bilirubin (mg/dL) | 0.7 (0.5-0.9) | 0.7 (0.5-1.0) | 0.6 (0.5-0.9) | **<0.001** |
| C reactive Protein (mg/L) | 6.3 (1.6-27.5) | 5.1 (1.1-24.0) | 8.3 (2.2-28.2) | **0.034** |
| Procalcitonin (ng/mL) | 0.05 (0.05-0.18) | 0.1 (0.1-0.2) | 0.1 (0.1-0.2) | 0.625 |
| NT-proBNP (pg/mL) | 1464.5 (420.8-3833.8) | 1252.5 (338.5-3041.3) | 1723.5 (473.8-5189.5) | **0.033** |
| Troponin Ultra (ng/L) | 0.11 (0.01-0.04) | 0.01 (0.01-0.03) | 0.01 (0.01-0.04) | **0.002** |
