# Supplementary material for: Sex-based differences in emergency department treatment times for acute ischaemic stroke: evidence from a large Italian cohort
Source: Eur Stroke J. 2026 May 11;11(5):aakag039. doi: 10.1093/esj/aakag039 (PMC13160415; doi:10.1093/esj/aakag039)
Supplement: aakag039_Supplemental_Files [file aakag039_supplemental_files.zip › Table_S5_aakag039.docx]

**Table S5.** Results of the comparison of the clinical characteristics of patients with ischemic stroke who underwent revascularization treatments and those who not.

|  | | | **Reperfusion therapy**  (n = 934) | **No reperfusion therapy**  (n = 3136) | **p-value** |
| --- | --- | --- | --- | --- | --- |
| **Demographics** | Age (years) | | 76.0 (65.0-83.0) | 77.0 (67.0-85.0) | **0.001** |
|  | Sex (Female) | | 464 (49.7%) | 1496 (47.7%) | 0.289 |
| **Triage** | Triage code | Emergency | 807 (86.4%) | 12261 (40.2%) | **<0.001** |
|  |  | Urgency | 119 (12.7%) | 1520 (48.5%) |  |
|  |  | Minor Urgency | 8 (0.9%) | 355 (11.3%) |  |
|  | ED waiting time before medical assessment (min) | | 5.0 (3.0-8.0) | 10.0 (5.0-25.0) | **<0.001** |
|  | ED waiting time>15 minutes | | 78 (8.4%) | 1133 (36.1%) | <0.001 |
| **Mode of ED arrival** | Emergency Medical Service | | 1708 (54.5%) | 735 (78.7%) | **<0.001** |
| **Onset to door times** | <3 hours | | 693 (74.2%) | 1282 (40.9%) | **<0.001** |
|  | 3-6 hours | | 165 (17.7%) | 634 (20.2%) |  |
|  | 6-12 hours | | 44 (4.7%) | 369 (11.8%) |  |
|  | 12-24 hours | | 9 (1.0%) | 267 (8.5%) |  |
|  | >24 hours | | 23 (2.5%) | 584 (18.6%) |  |
| **ED time metrics** | ED length of stay (h) | | 2.0 (1.3-3.4) | 7.1 (2.6-24.0) | **<0.001** |
|  | Door-to-CT scan time (min) | | 22.0 (14.0-35.0) | NA | NA |
|  | Door-to-needle time (min) | | 52.0 (39.0-68.0) | NA | NA |
|  | Door-to-groin time (min) | | 125.0 (101.0-150.75) | NA | NA |
| **Vitals**  **(ED admission)** | Heart rate (bpm) | | 78.0 (68.0-89.0) | 80.0 (70.0-91.0) | **<0.001** |
|  | Systolic blood pressure (mmHg) | | 150.0 (130.0-170.0) | 150.0 (130.0-168.0) | 0.918 |
|  | Diastolic blood pressure (mmHg) | | 84.0 (75.0-95.0) | 84.0 (73.0-95.0) | 0.858 |
|  | SaO2 (%) | | 97.0 (95.0-98.0) | 97.0 (95.0-98.0) | 0.738 |
| **Neurological symptoms**  **(ED admission)** | NIHSS | | 12.0 (7.0-18.0) | 3.5 (1.3-6.8) | **<0.001** |
|  | Aphasia | | 642 (68.7%) | 292 (31.3%) | **<0.001** |
|  | Motor impairment | | 617 (66.1%) | 2128 (67.9%) | 0.304 |
|  | Sensory impairment | | 29 (3.1%) | 296 (9.4%) | **<0.001** |
|  | Headache | | 29 (3.1%) | 259 (8.3%) | **<0.001** |
|  | Epileptic seizure | | 23 (2.5%) | 144 (4.6%) | **0.004** |
|  | Confusion/Disorientation | | 87 (9.3%) | 575 (18.3%) | **<0.001** |
|  | Impaired consciousness | | 175 (18.7%) | 451 (14.4%) | **0.001** |
|  | Dizziness | | 15 (1.6%) | 226 (7.2%) | **<0.001** |
|  | Malaise | | 66 (7.1%) | 426 (13.6%) | **<0.001** |
|  | Gait disturbances | | 26 (2.8%) | 300 (9.6%) | **<0.001** |
|  | Syncope | | 46 (4.9%) | 220 (7.0%) | **0.023** |
| **Comorbidities** | Charlson Comorbidity Index | | 4.0 (3.0-6.0) | 4.0 (2.0-6.0) | 0.304 |
|  | Previous AMI or CAD | | 231 (24.7%) | 790 (25.2%) | 0.776 |
|  | Atrial fibrillation | | 286 (30.6%) | 730 (23.3%) | **<0.001** |
|  | Heart failure | | 176 (18.8%) | 758 (24.2%) | **0.001** |
|  | Arterial hypertension | | 536 (57.4%) | 1538 (49.0%) | **<0.001** |
|  | Peripheral artery disease | | 214 (22.9%) | 770 (24.6%) | 0.304 |
|  | Previous TIA/Stroke | | 666 (71.3%) | 1926 (61.4%) | **<0.001** |
|  | Major neurocognitive disorder | | 18 (1.9%) | 193 (6.2%) | **<0.001** |
|  | COPD | | 30 (3.2%) | 149 (4.8%) | **0.044** |
|  | Connective tissue disease | | 6 (0.6%) | 30 (1.0%) | 0.368 |
|  | Liver disease | | 5 (0.5%) | 34 (1.1%) | 0.131 |
|  | Diabetes | | 143 (15.3%) | 603 (19.2%) | **0.007** |
|  | Kidney failure | | 130 (13.9%) | 497 (15.8%) | 0.152 |
|  | Solid cancer | | 38 (4.1%) | 242 (7.7%) | **<0.001** |
|  | Metastasis | | 6 (0.6%) | 62 (2.0%) | **0.005** |
|  | HIV + | | 0 (0.0%) | 5 (0.2%) | 0.222 |
| **Outcomes** | Hospitalization | | 924 (98.9%) | 2737 (87.3%) | **<0.001** |
|  | Hospitalization in Neurology department | | 613 (65.6%) | 1795 (54.4%) | **<0.001** |
|  | Hospitalization length (days) | | 7.5 (4.8-14.2) | 7.5 (4.5-12.5) | **0.012** |
|  | Need of mechanical ventilation | | 157 (16.8%) | 153 (4.9%) | **<0.001** |
|  | Death | | 95 (10.2%) | 382 (12.2%) | 0.094 |

*Abbreviations: ED, Emergency Department; min, minutes; h, hours; bpm, beats per minutes; mmHg, millimetres of mercury; SaO2, Oxygen Saturation; NIHSS, National Institutes of Health Stroke Scale; AMI, Acute Myocardial Infarction; CAD, Coronary Artery Disease; TIA, Transient Ischemic Attack; COPD, Chronic Obstructive Pulmonary Disease; HIV, Human Immunodeficiency Virus*
