# Supplementary material for: Sex-based differences in emergency department treatment times for acute ischaemic stroke: evidence from a large Italian cohort
Source: Eur Stroke J. 2026 May 11;11(5):aakag039. doi: 10.1093/esj/aakag039 (PMC13160415; doi:10.1093/esj/aakag039)
Supplement: aakag039_Supplemental_Files [file aakag039_supplemental_files.zip › Table_S6_aakag039.docx]

**Table S6.** Sex-specific comparison of reperfusion therapies and treatment time metrics until and after 2018

|  | **2015-2018** | | |  | **2019-2022** | | |
| --- | --- | --- | --- | --- | --- | --- | --- |
|  | **Female**  **(n = 999)** | **Male**  **(n = 994)** | **p-value** |  | **Female**  **(n = 1116)** | **Male**  **(n = 961)** | **p-value** |
| Intravenous Thrombolysis | 168 (16.8%) | 157 (15.8%) | 0.537 |  | 141 (14.7%) | 179 (16%) | 0.389 |
| Endovascular Treatment | 76 (7.6%) | 70 (7.0%) | 0.628 |  | 166 (17.3%) | 154 (13.8%) | **0.029** |
| Reperfusion Therapies | 206 (20.6%) | 194 (19.5%) | 0.539 |  | 258 (26.8%) | 276 (24.7%) | 0.271 |
| Door-to-needle time (min) | 53.0 (40.5-71.5) | 50.5 (39.0-67.0) | 0.368 |  | 54.0 (39.0-71.5) | 49.0 (37.5-61.8) | **0.032** |
| Door-to-groin time (min) | 121.0 (101.3-163.0) | 119.5 (87.5-141.8) | 0.145 |  | 127.0 (102.0-157.0) | 125.0 (105.0-155.0) | 0.857 |
| Door-to-CT time (min) | 18.0 (11.0-33.0) | 17.0 (10.0-28.0) | 0.237 |  | 25.0 (18.0-41.0) | 25.0 (17.036.0) | 0.380 |
