# Supplementary material for: Sex-based differences in emergency department treatment times for acute ischaemic stroke: evidence from a large Italian cohort
Source: Eur Stroke J. 2026 May 11;11(5):aakag039. doi: 10.1093/esj/aakag039 (PMC13160415; doi:10.1093/esj/aakag039)
Supplement: aakag039_Supplemental_Files [file aakag039_supplemental_files.zip › Table_S7_aakag039.docx]

**Table S7.** Results of the multivariable logistic analysis for intravenous thrombolysis.

| **Parameter** | **OR (95%CI)** | **p-value** | **VIF** |
| --- | --- | --- | --- |
| Sex | 1.119 (0.790 – 1.586) | 0.525 | 1.114 |
| Age | 1.006 (0.993 – 1.020) | 0.359 | 1.237 |
| NIHSS | 0.977 (0.954 – 1.000) | **0.046** | 1.157 |
| Onset to door time | 0.524 (0.421 – 0.653) | **<0.001** | 1.149 |
| Emergency Medical Service | 0.934 (0.598 – 1.460) | 0.764 | 1.141 |
| Triage codes | 2.165 (1.251 – 3.744) | **0.006** | 1.185 |
| Diabetes | 1.110 (0.695 – 1.773) | 0.662 | 1.047 |
| Cancer | 0.806 (0.379 – 1.715) | 0.576 | 1.014 |
| Arterial hypertension | 1.210 (0.869 – 1.686) | 0.259 | 1.075 |
| Atrial fibrillation | 0.468 (0.331 – 0.661) | **<0.001** | 1.168 |
| Major neurocognitive disorder | 0.701 (0.245 – 2.010) | 0.509 | 1.026 |
| Previous stroke/TIA | 1.587 (1.099 – 2.290) | **0.014** | 1.040 |

*Abbreviations: OR, Odds Ratio; CI, Confidence Interval; VIF, Variance Inflation Factor; NIHSS, National Institutes of Health Stroke Scale; TIA, Transient Ischemic Attack. *reference value.*
