# Supplementary material for: Sex-based differences in emergency department treatment times for acute ischaemic stroke: evidence from a large Italian cohort
Source: Eur Stroke J. 2026 May 11;11(5):aakag039. doi: 10.1093/esj/aakag039 (PMC13160415; doi:10.1093/esj/aakag039)
Supplement: aakag039_Supplemental_Files [file aakag039_supplemental_files.zip › Table_S8_aakag039.docx]

**Table S8.** Results of the multivariable logistic analysis for endovascular treatment.

| **Parameter** | **OR (95%CI)** | **p-value** | **VIF** |
| --- | --- | --- | --- |
| Sex | 0.922 (0.666 – 1.276) | 0.624 | 1.114 |
| Age | 0.985 (0.973 – 0.997) | **0.014** | 1.237 |
| NIHSS | 1.119 (1.090 – 1.148) | **<0.001** | 1.157 |
| Onset to door times | 1.661 (1.328 – 2.078) | **<0.001** | 1.149 |
| EMS | 0.976 (0.641 – 1.486) | 0.909 | 1.141 |
| Triage code | 0.498 (0.304 – 0.817) | **0.006** | 1.185 |
| Diabetes | 0.726 (0.475 – 1.109) | 0.138 | 1.047 |
| Cancer | 1.147 (0.556 – 2.365) | 0.710 | 1.014 |
| Arterial hypertension | 1.261 (0.913 – 1.740) | 0.159 | 1.075 |
| Atrial fibrillation | 1.893 (1.333 – 2.689) | **<0.001** | 1.168 |
| Major neurocognitive disorder | 0.644 (0.225 – 1.847) | 0.413 | 1.026 |
| Previous stroke/TIA | 0.593 (0.414 – 0.850) | **0.004** | 1.040 |

*Abbreviations: OR, Odds Ratio; CI, Confidence Interval; VIF, Variance Inflation Factor; NIHSS, National Institutes of Health Stroke Scale; TIA, Transient Ischemic Attack. *reference value.*
