# Supplementary material for: Sex-based differences in emergency department treatment times for acute ischaemic stroke: evidence from a large Italian cohort
Source: Eur Stroke J. 2026 May 11;11(5):aakag039. doi: 10.1093/esj/aakag039 (PMC13160415; doi:10.1093/esj/aakag039)
Supplement: aakag039_Supplemental_Files [file aakag039_supplemental_files.zip › Table_S9_aakag039.docx]

**Table S9.** Results of the multivariable linear regression for Door-to-needle time.

| **Parameter** | **B (95%CI)** | **p-value** | **VIF** |
| --- | --- | --- | --- |
| Sex | -8.242 (-15.453 - -1.031) | **0.025** | 1.132 |
| Age | -0.008 (-0.0284 – 0.267) | 0.952 | 1.259 |
| NIHSS | 0.890 (-0.405 – 0.467) | 0.890 | 1.149 |
| Onset to door time | 6.518 (0.913 – 12.123) | **0.023** | 1.193 |
| Emergency Medical Service | 4.496 (-4.851 – 13.842) | 0.345 | 1.201 |
| Triage codes | 42.242 (32.575 – 51.908) | **<0.001** | 1.253 |
| Diabetes | 0.668 (-8.286 – 9.622) | 0.884 | 1.056 |
| Cancer | 13.367 (-3.202 – 29.935) | 0.114 | 1.019 |
| Arterial hypertension | 2.486 (-4.740 – 9.712) | 0.499 | 1.096 |
| Atrial fibrillation | 0.636 (-7.775 – 9.048) | 0.882 | 1.177 |
| Major neurocognitive disorder | 3.375 (-20.491 – 27.242) | 0.781 | 1.040 |
| Previous stroke/TIA | 1.778 (-6.377 – 9.933) | 0.669 | 1.060 |

*Abbreviations: OR, Odds Ratio; CI, Confidence Interval; VIF, Variance Inflation Factor; NIHSS, National Institutes of Health Stroke Scale; TIA, Transient Ischemic Attack. *reference value.*
